# On the infinite Borwein product raised to a positive real power

**DOI:** 10.1007/s11139-021-00519-3

**Published:** 2021-11-02

**Authors:** Michael J. Schlosser, Nian Hong Zhou

**Affiliations:** 1 Fakultät für Mathematik, Universität Wien, Oskar-Morgenstern-Platz 1, 1090 Vienna, Austria; 2 School of Mathematics and Statistics, Guangxi Normal University, No. 1 Yanzhong Road, Yanshan District, Guilin, 541006 Guangxi People’s Republic of China

**Keywords:** Infinite Borwein product, Sign pattern, Asymptotics, Positivity, Circle method, Vanishing of coefficients, Primary 11P55, Secondary 11F03, 11F30, 26D20

## Abstract

In this paper, we study properties of the coefficients appearing in the *q*-series expansion of $\prod _{n\ge 1}[(1-q^n)/(1-q^{pn})]^\delta$, the infinite Borwein product for an arbitrary prime *p*, raised to an arbitrary positive real power $\delta$. We use the Hardy–Ramanujan–Rademacher circle method to give an asymptotic formula for the coefficients. For $p=3$ we give an estimate of their growth which enables us to partially confirm an earlier conjecture of the first author concerning an observed sign pattern of the coefficients when the exponent $\delta$ is within a specified range of positive real numbers. We further establish some vanishing and divisibility properties of the coefficients of the cube of the infinite Borwein product. We conclude with an Appendix presenting several new conjectures on precise sign patterns of infinite products raised to a real power which are similar to the conjecture we made in the $p=3$ case.

## Introduction and statement of results

Let *q* be a complex number with $0<|q|<1$. Define

1.1 $$\begin{aligned} f(q)=\prod _{n\ge 1}\frac{1}{1-q^n}, \end{aligned}$$

and, for *p* being a prime,

1.2 $$\begin{aligned} G_p(q)=\frac{f(q^p)}{f(q)}. \end{aligned}$$

We shall call $G_p(q)$ the *infinite Borwein product*. It is well known that *f*(*q*) is the generating function for the number of unrestricted partitions *p*(*n*), that is

$$\begin{aligned} f(q)=\sum _{n\ge 0}p(n)q^n. \end{aligned}$$

Using the modularity of *f*(*q*), Hardy and Ramanujan [11] and Rademacher [16] proved that

1.3 $$\begin{aligned} p(n)=\frac{1}{\pi \sqrt{2}} \sum _{k\ge 0}k^{1/2}\sum _{\begin{array}{c} h\!\!\pmod {k}\\ \gcd (h,k)=1 \end{array}}\omega _{h,k}\mathrm{{e}}^{-\frac{2\pi \mathrm ihn}{k}} \frac{\,d}{\,dn}\frac{\sinh \Big (\frac{\pi }{k}\sqrt{\frac{2}{3}(n-1/24)}\Big )}{\sqrt{n-1/24}}, \end{aligned}$$

for all integers $n\ge 1$. Here and throughout this paper,

1.4 $$\begin{aligned} \omega _{h,k}=\mathrm{{e}}^{\pi \mathrm is(h,k)}, \end{aligned}$$

with *s*(*h*, *k*) being the Dedekind sum

1.5 $$\begin{aligned} s(h,k)=\sum _{1\le j<k}\bigg (\frac{j}{k}- \left\lfloor \frac{j}{k}\right\rfloor - \frac{1}{2}\bigg )\bigg (\frac{jh}{k}- \left\lfloor \frac{jh}{k}\right\rfloor -\frac{1}{2}\bigg ). \end{aligned}$$

According to Andrews [1], P. Borwein considered the *q*-series expansion

1.6 $$\begin{aligned} G_{p}(q)=\sum _{n\ge 0}c_{p}(n)q^n, \end{aligned}$$

as part of an unpublished study of modular forms. While it is clear from (1.2) that $G_p(q)^{-1}$ is the generating function for partitions into parts that are not a multiple of *p*, and thus has non-negative coefficients, the coefficients $c_p(n)$ in (1.6) have different signs. Andrews [1, Theorem 2.1] proved the following result, and noted that Garvan and Borwein have a different proof in unpublished work of 1990.

### Theorem 1

For all primes *p*, $c_p(n)$ and $c_p(n+p)$ have the same sign for each $n\ge 0$, i.e.,

$$\begin{aligned} c_{p}(n)c_{p}(n+p)\ge 0, \end{aligned}$$

for each $$n\ge 0$$.

We say that the coefficients $c_p(n)$ have a sign pattern of period *p*.

In September 2019, as a result of experimentation using computer algebra, the first author of the present paper presented a conjecture [20, Conjecture 1] in a tribute dedicated to Richard Askey. We reproduce this conjecture in Conjecture 2 below; one of the main results of this paper is a partial affirmation of it, see Corollary 5.

### Conjecture 2

Let $\delta$ be a real number satisfying

$$\begin{aligned} 0.227998127341\ldots \approx \frac{9-\sqrt{73}}{2}\le \delta \le 1\quad \text {or} \quad 2\le \delta \le 3. \end{aligned}$$

Then the series $A^{(\delta )}(q)$, $B^{(\delta )}(q)$, $C^{(\delta )}(q)$ appearing in the dissection

$$\begin{aligned} G_3(q)^\delta = A^{(\delta )}(q^3)-qB^{(\delta )}(q^3)-q^2C^{(\delta )}(q^3) \end{aligned}$$

are power series in *q* with non-negative real coefficients.

With other words, for the exponent $\delta$ within the specified range of real numbers the *q*-series coefficients of $G_3(q)^\delta$ exhibit the sign pattern $+--$.

We present several similar conjectures on precise sign patterns for other infinite products raised to a power within specified ranges of real numbers in Appendix 1.

The validity of Conjecture 2 for $\delta =1$ is known and easy to prove by using Jacobi’s triple product identity, see e.g. [22]. For $\delta =3$, we actually have a result for any prime *p*, not only for $p=3$, see Theorem 6.

It is actually not difficult to explain why the condition $\delta \in [\frac{9-\sqrt{73}}{2},1]\cup [2,3]$ (leaving out the trivial case $\delta =0$) is *necessary* for the sign-pattern $+--$ to hold. In fact, we have the Taylor series expansion (which is routine to compute using any computer algebra system)

$$\begin{aligned} G_3(q)^\delta&=1-\delta q+\frac{\delta (\delta -3)}{2} q^2- \frac{\delta (\delta ^2-9\delta +2)}{6} q^3 +\frac{\delta (\delta ^3-18\delta ^2+35\delta -42)}{24} q^4\\&\quad \times \, -\frac{\delta (\delta -1)(\delta -2)(\delta -3)(\delta -24)}{120} q^5 +O(q^6). \end{aligned}$$

For the sign pattern $+--$ to hold, first of all the coefficient of $q^1$ in $G_3(q)^\delta$ should be non-positive. This implies $\delta >0$. (We excluded the trivial case $\delta =0$ in the first place.) The coefficient of $q^2$ should be non-positive as well. This forces $0<\delta \le 3$. We turn to the coefficient of $q^3$. The two roots of $\delta ^2-9\delta +2$ are $\frac{9\pm \sqrt{73}}{2}$ and it is easy to see that the coefficient of $q^3$ can only be non-negative if $\frac{9-\sqrt{73}}{2}\le \delta \le \frac{9+\sqrt{73}}{2}$. Since $\delta \le 3$ (from before) we have reached the point that we need $\frac{9-\sqrt{73}}{2}\le \delta \le 3$. Finally, for the coefficient of $q^5$ (we don’t need to consider the coefficient of $q^4$ here) to be non-positive we obviously need to exclude $1<\delta <2$. Altogether we have explained the necessity of the specified range of real numbers for $\delta$. The surprising fact is that this range is also (conjectured to be) *sufficient* for all of the coefficients to satisfy the sign pattern $+--$.

While Conjecture 2 concerns a statement about a sign-pattern that holds from the first coefficient on for suitably restricted $\delta >0$, we actually believe that the sign pattern $+--$ holds for any $\delta >0$ in an asymptotic sense, namely from the *n*-th coefficient on, where *n* is an integer depending on $\delta$.

This serves as our motivation to apply an asymptotic approach towards settling Conjecture 2 where we initially just assume $\delta >0$ (not further restricted), and only later restrict $\delta$ to be within specified intervals when desired. To achieve our goal we shall employ the Hardy–Ramanujan circle method perfected by Rademacher [16] (see also [17, Chapter 14]). This will enable us to give an asymptotic formula for the *q*-series coefficients $c_p^{(\delta )}(n)$ appearing in the infinite Borwein product raised to a real power $\delta >0$, i.e. of

1.7 $$\begin{aligned} G_p(q)^{\delta }=\sum _{n\ge 0}c_p^{(\delta )}(n)q^n, \end{aligned}$$

where *p* is any prime (not necessarily $p=3$). We shall refer to the $c_p^{(\delta )}(n)$ as *Borwein coefficients*.

At this point it is appropriate to mention that the use of asymptotic machinery to prove positivity results (including sign patterns) for the coefficients appearing in infinite *q*-products is quite established and known to be efficient. In particular, Richmond and Szekeres [19], making heavy use of results of Iseki [13, 14], employed the Rademacher circle method to prove the sign pattern of the Göllnitz–Gordon continued fraction. Recently, Chern [7, 8] established the asymptotics of the coefficients of any finite product of Dedekind eta functions, and similarly the asymptotics of the coefficients of *q*-products satisfying modular symmetries. His methods are very similar to those we use in the present paper but we consider arbitrary real powers of the infinite products (and our applications are of a different, more analytic nature). We would also like to mention that C. Wang [23] recently utilized asymptotic machinery to settle the famous first Borwein Conjecture (cf. [1]) which is a statement about the coefficients appearing in a sequence of *finite* products. Some related open conjectures about sequences of infinite products were recently raised by Bhatnagar and the first author in [4], however, no attempt was made there to attack the conjectures by asymptotic machinery or by other means.

In order to state our results we recall the definition of the modified Bessel function of the first kind $I_1(z)$ given by

1.8 $$\begin{aligned} I_1(z):=\sum _{n\ge 0}\frac{1}{n!(n+1)!}\Big (\frac{z}{2}\Big )^{2n+1}, \end{aligned}$$

cf. [2, p. 222, Eq. (4.12.2)], which is an entire function. Its integral representation is

1.9 $$\begin{aligned} I_1(z)=\frac{(z/2)}{2\pi \mathrm i}\int _{1-\mathrm i\infty }^{1+\mathrm i\infty } \mathrm{{e}}^{w+z^2/4w}w^{-2}\,\mathrm dw, \end{aligned}$$

cf. [2, p. 236, Exercise 13].

For any prime *p*, we have the following asymptotic formula (of arbitrary positive integer order *N*) for the Borwein coefficients $c_p^{(\delta )}(n)$, where $\delta$ is within a specified range of positive real numbers depending on *p*. (Recall that, according to (1.4) and (1.5), $\omega _{h,k}$ denotes certain exponentials of Dedekind sums.)

### Theorem 3

Let $\delta \in (0,24/(p-1)]$ and let $N\in {\mathbb {N}}$. For each integer $n\ge 1$ we have

$$\begin{aligned} c_p^{(\delta )}(n)&=\frac{2\pi \delta ^{1/2}}{\sqrt{\frac{24n}{p-1}-\delta }} \sum _{1\le k\le N }A_{pk}^{(\delta )}(n)I_1\Bigg (\frac{(p-1)\pi }{6pk} \sqrt{\delta \bigg (\frac{24n}{p-1}-\delta \bigg )}\Bigg )\\*&\quad \;+\mathrm{{e}}^{\frac{(24n-(p-1)\delta )\pi }{6p^2N^2}}E_{p,N}^{(\delta )}(n), \end{aligned}$$

where

$$\begin{aligned} A_k^{(\delta )}(n)=\frac{1}{k}\sum _{\begin{array}{c} 0\le h<k\\ \gcd (h,k)=1 \end{array}} \Big (\omega _{h,k}^{-1}\omega _{h,\frac{k}{p}}\Big )^{\delta } \mathrm{{e}}^{-\frac{2\pi \mathrm ihn}{k}}. \end{aligned}$$

Further, the error term $E_{p,N}^{(\delta )}(n)$ satisfies the bound

$$\begin{aligned} \Big |E_{p,N}^{(\delta )}(n)\Big |&\le \frac{(p-1)\mathrm{{e}}^{\frac{(p-1)\pi \delta }{12}} }{p^2}\Big (\pi \sqrt{2}-2+ 2f\big (\mathrm{{e}}^{-6\pi }\big )^{\delta }f\big (\mathrm{{e}}^{-2\pi }\big )^{\delta }\Big )\\*&\quad \;+ \frac{ 2(p-1)\cdot \mathrm{{e}}^{-\frac{\pi (p-1)\delta }{12p}}}{p^{1-\delta /2}}f\Big (\mathrm{{e}}^{-\frac{2\pi }{p}}\Big )^{\delta } f\big (\mathrm{{e}}^{-2\pi }\big )^{\delta }. \end{aligned}$$

### Remark 1

Throughout this paper, $z^{a}:=\mathrm{{e}}^{a\log z}$ and the logarithms are always understood to assume their principal values, that is $\arg (z)\in [-\pi , \pi )$.

### Remark 2

The right-hand side of the inequality for $\big |E_{p,N}^{(\delta )}(n)\big |$ in Theorem 3 is independent from *N* and *n*, thus the error term $E_{p,N}^{(\delta )}(n)$ is *O*(1). By using a similar argument to that of Rademacher and Zuckerman in their proof of [18, Theorem 1], we can extend the specified region for $\delta$ in Theorem 3 (which is $\delta \in (0,24/(p-1)]$) to all $\delta >0$, still with an *O*(1) error term. However, the expressions for the main term and the effective error term are then more complicated. Since we are mainly interested in the asymptotics in certain confined regions (after all, our main aim concerns the development of tools to understand and tackle concrete observations such as those in Conjecture 2 and similar conjectures in Appendix 1), we leave the details of using Rademacher and Zuckerman’s method to to extend the range of $\delta$ to all positive reals to the interested reader.

Focusing on the case $p=3$, we can use Theorem 3 to give the following growth estimate for the Borwein coefficients $c_3^{(\delta )}(n)$ for $\delta$ within a specified range.

### Theorem 4

Let $\delta \in [0.227,3]$, and define

$$\begin{aligned} {\hat{c}}_3^{(\delta )}(n)&=\frac{2\pi \delta ^{1/2}}{3\sqrt{12n-\delta }}\, I_1\Big (\frac{\pi }{9}\sqrt{\delta \left( 12n-\delta \right) }\Big ),\\* L_{\delta ,n}&=\frac{\pi }{18}\sqrt{\delta \left( 12n-\delta \right) }, \end{aligned}$$

and

$$\begin{aligned} w(\delta )=\frac{1}{2}\log \bigg (\frac{1}{\delta }\bigg )+ \frac{0.736\,\big (1.689^{\delta }\big (1.222+1.002^{\delta }\big )+ 3\cdot 1.692^{\delta }\big )}{\delta }+0.119. \end{aligned}$$

Then we have for all $n\in {\mathbb {N}}$ the inequality

$$\begin{aligned} \Bigg |\frac{c_3^{(\delta )}(n)}{{\hat{c}}_3^{(\delta )}(n)} - \cos \bigg (\frac{\pi \delta }{18}+\frac{2\pi n}{3}\bigg )\Bigg |\le \frac{L_{\delta ,n} w(\delta )+L_{\delta ,n}\log L_{\delta ,n} +2 I_1(L_{\delta ,n})}{I_1(2L_{\delta ,n})}. \end{aligned}$$

We obtained the numerical constants appearing in the expression for $w(\delta )$ with the aid of *Mathematica*; a strengthening of the result with a higher precision of the involved constants is a question of computational resources (suitable software, running time and memory). In principle, Theorem 3 would even enable us to give precise growth estimates for the Borwein coefficients $c_3^{(\delta )}(n)$ for $\delta$ within the larger range $[\epsilon ,12]$ where $\epsilon$ is any given positive real number. Now our experimentation using *Mathematica* showed that the computations converge considerably faster for $\epsilon \le \delta \le 3$ where $\epsilon$ is not much less than $\frac{9-\sqrt{73}}{2}$ than outside this region (which is not a big surprise in view of Conjecture 2). Therefore, for practical computational reasons we took $\epsilon =0.227$ and restricted the initial range (0, 12] (coming from the $p=3$ case of Theorem 3) to the range [0.227, 3] which is still larger than the range for $\delta$ specified in Conjecture 2, namely $[\frac{9-\sqrt{73}}{2},1]\cup [2,3]$ which is the range we mainly care about. At this point we would like to remind the reader that Conjecture 2 concerns an assertion about the *precise* behavior of the coefficients of a series while Theorem 4 (and the following Corollary 5) concerns their *asymptotic* behavior.

Finally, we are able to partially affirm Conjecture 2 (again, with numerical constants obtained with the aid of *Mathematica*) in the following form:

### Corollary 5

For all integers $n\ge 158$ and for all $\delta$ such that

$$\begin{aligned} 0.227\le \delta \le 2.9999, \end{aligned}$$

we have

$$\begin{aligned} c_3^{(\delta )}(n)\,c_3^{(\delta )}(n+3)>0. \end{aligned}$$

### Remark 3

While Corollary 5 only partially affirms Conjecture 2, it also gives information about the cases when $0.227\le \delta <\frac{9-\sqrt{73}}{2}$ and $1<\delta <2$ (not covered by the conjecture). In these cases case the corollary tells us that the Borwein coefficients $c_3^{(\delta )}(n)$ satisfy the respective sign pattern for large enough *n* (namely $n\ge 158$).

For the exponent $\delta =3$ we actually have the following result for any prime *p* which is a cubic analogue of Theorem 1:

### Theorem 6

For all primes *p*, $c_p^{(3)}(n)$ and $c_p^{(3)}(n+p)$ have the same sign for each $n\ge 0$, i.e.,

$$\begin{aligned} c_{p}^{(3)}(n)c_{p}^{(3)}(n+p)\ge 0, \end{aligned}$$

for each $$n\ge 0$$.

The proof is given in Sect. 4.

Our paper is organized as follows: In Sect. 2 we prove Theorem 3, thus establish an asymptotic formula for the Borwein coefficients $c_p^{(\delta )}(n)$, for any prime *p*. In Sect. 3 we turn to the $p=3$ case. We prove Theorem 4 there, which provides us with a useful estimate for the growth of the coefficients $c_3^{(\delta )}(n)$. This allows us to prove Corollary 5. In Sect. 4, which is of independent interest, we prove some results that include vanishing and divisibility properties for the Borwein coefficients of the cube of the infinite Borwein product. Finally, in Appendix 1 we present several new conjectures on precise sign patterns of infinite Borwein products and other products raised to a real power, which are similar to Conjecture 2.

## The proof of Theorem 3

Our proof is in two steps. In the first step we establish a modular transformation for the generating function $G_{p}(q)^{\delta }$. In the next step we follow Rademacher’s method and use the modular transformation to obtain an expansion for the Borwein coefficients $c_p^{(\delta )}(n)$.

Recall that *f*(*q*) and $G_p(q)$ were defined in (1.1) and (1.2), respectively. Further, we would like to remind the reader about the notation $\omega _{h,k}$ used for certain exponentials involving Dedekind sums, see (1.4) and (1.5), which prominently appear in the Hardy–Ramanujan–Rademacher circle method (see (1.3)).

### Modular transformation for the generating function

#### Proposition 7

Let $h,k\in {\mathbb {Z}}$ such that $k>0$ and $\gcd (h,k)=1$. Let $d=\gcd (p,k)$, and let $h'$ and $h_d'$ be solutions of the congruences

$$\begin{aligned} hh'&\equiv 1 ~(\bmod ~k)\\ \end{aligned}$$

and

$$\begin{aligned} (hp/d)h_d'&\equiv 1 ~(\bmod ~{k/d}). \end{aligned}$$

Then, for all $\delta \in {\mathbb {R}}$ and $\mathfrak {R}(z)>0$ we have

$$\begin{aligned} G_{p}\Big (\mathrm{{e}}^{\frac{2\pi \mathrm ih}{k}-\frac{2\pi z}{k^2}}\Big )^{\delta }&=\Big (\frac{p}{d}\Big )^{\frac{\delta }{2}} \Big (\omega _{h,k}^{-1}\omega _{\frac{ph}{d},\frac{k}{d}}\Big )^{\delta } \exp \bigg (\frac{\pi \delta (d^2-3)}{36z}-\frac{\pi \delta z}{6k^2}\bigg )\\&\quad \times \,{\hat{G}}_{p}\Big (h,k; \mathrm{{e}}^{-\frac{2\pi }{z}}\Big )^{\delta }, \end{aligned}$$

where

$$\begin{aligned} {\hat{G}}_{p}\Big (h,k; \mathrm{{e}}^{-\frac{2\pi }{z}}\Big ) =f\bigg (\mathrm{{e}}^{\frac{2\pi \mathrm idh_d'}{k}-\frac{2\pi d^2}{pz}}\bigg ) f\Big (\mathrm{{e}}^{\frac{2\pi \mathrm ih'}{k}-\frac{2\pi }{z}}\Big )^{-1}. \end{aligned}$$

#### Proof

Notice that the functions occurring in the statement of the proposition are all holomorphic on $\mathfrak {R}(z)>0$. We just need to show that the transformation holds for all positive real *z*, then the full transformation follows by analytic continuation. For $z>0$, from Hardy and Ramanujan [11, Lemma 4.31], we have

$$\begin{aligned} f\Big (\mathrm{{e}}^{\frac{2\pi \mathrm ih}{k}-\frac{2\pi z}{k^2}}\Big ) =\omega _{h,k}\Big (\frac{z}{k}\Big )^{\frac{1}{2}} \exp \bigg (\frac{\pi }{12z}-\frac{\pi z}{12k^2}\bigg ) f\bigg (\mathrm{{e}}^{\frac{2\pi \mathrm ih'}{k}-\frac{2\pi }{z}}\bigg ), \end{aligned}$$

where $\omega _{h,k}=\mathrm{{e}}^{\pi \mathrm is(h,k)}$, and *s*(*h*, *k*) is defined by (1.5). Taking into account $d=\gcd (p,k)$, we thus have

$$\begin{aligned} G_{p}\Big (\mathrm{{e}}^{\frac{2\pi \mathrm ih}{k}-\frac{2\pi z}{k^2}}\Big )= & {} f\bigg (\mathrm{{e}}^{\frac{2\pi \mathrm ih(p/d)}{k/d}-\frac{2\pi (pz/d^2)}{(k/d)^2}}\bigg ) f\Big (\mathrm{{e}}^{\frac{2\pi \mathrm ih}{k}-\frac{2\pi z}{k^2}}\Big )^{-1}\\= & {} \omega _{\frac{hp}{d},\frac{k}{d}}\bigg (\frac{pz/d^2}{k/d}\bigg )^{\frac{1}{2}} \exp \bigg (\frac{\pi }{12(pz/d^2)}-\frac{\pi (pz/d^2)}{12(k/d)^2}\bigg ) f\bigg (\mathrm{{e}}^{\frac{2\pi \mathrm ih_d'}{(k/d)}-\frac{2\pi }{pz/d^2}}\bigg )\\&\quad \times \,\omega _{h,k}^{-1}\Big (\frac{z}{k}\Big )^{-\frac{1}{2}} \exp \bigg (-\frac{\pi }{12z}+\frac{\pi z}{12k^2}\bigg ) f\bigg (\mathrm{{e}}^{\frac{2\pi \mathrm ih'}{k}-\frac{2\pi }{z}}\bigg )^{-1}\\= & {} \omega _{h,k}^{-1}\omega _{\frac{hp}{d},\frac{k}{d}} \Big (\frac{p}{d}\Big )^{\frac{1}{2}} \exp \bigg (\frac{\pi (d^2-p)}{12pz}-\frac{(p-1)\pi z}{12k^2}\bigg ) {\hat{G}}_{p}\Big (h,k; \mathrm{{e}}^{-\frac{2\pi }{z}}\Big ). \end{aligned}$$

From this we obtain for all $\delta >0$,

$$\begin{aligned} G_{p}\Big (\mathrm{{e}}^{\frac{2\pi \mathrm ih}{k}-\frac{2\pi z}{k^2}}\Big )^{\delta }&=\Big (\omega _{h,k}^{-1}\omega _{\frac{hp}{d},\frac{k}{d}} {\hat{G}}_{p}\Big (h,k; \mathrm{{e}}^{-\frac{2\pi }{z}}\Big )\Big )^{\delta }\\*&\quad \;\times \Big (\frac{p}{d}\Big )^{\frac{\delta }{2}} \exp \bigg (\frac{\delta (d^2-p)\pi }{12pz} -\frac{\delta (p-1)\pi z}{12k^2}\bigg ). \end{aligned}$$

It is obvious that $G_{p}\Big (\mathrm{{e}}^{\frac{2\pi \mathrm ih}{k}-\frac{2\pi z}{k^2}}\Big )^\delta$ can be analytically extended to a single valued analytic function on the right half plane $\mathfrak {R}(z)>0$. So the function on the right-hand side of the equation above has the same properties. Furthermore, one can find a small open set $\Omega$ on the right half plane $\mathfrak {R}(z)>0$ such that

$$\begin{aligned} \arg \Big (\omega _{h,k}^{-1}\omega _{\frac{hp}{d},\frac{k}{d}} \Big ) + \arg \Big ({\hat{G}}_{p}\Big (h,k; \mathrm{{e}}^{-\frac{2\pi }{z}}\Big )\Big ) \in [-\pi ,\pi ], \end{aligned}$$

for all $z\in \Omega$. This implies that if $z\in \Omega$ then

$$\begin{aligned} G_{p}\Big (\mathrm{{e}}^{\frac{2\pi \mathrm ih}{k}-\frac{2\pi z}{k^2}}\Big )^{\delta }&=\Big (\omega _{h,k}^{-1}\omega _{\frac{hp}{d},\frac{k}{d}}\Big )^{\delta } {\hat{G}}_{p}\Big (h,k; \mathrm{{e}}^{-\frac{2\pi }{z}}\Big )^{\delta }\\*&\quad \;\times \Big (\frac{p}{d}\Big )^{\frac{\delta }{2}} \exp \bigg (\frac{\delta (d^2-p)\pi }{12pz}- \frac{\delta (p-1)\pi z}{12k^2}\bigg ). \end{aligned}$$

Finally, noticing that ${\hat{G}}_{p}\Big (h,k; \mathrm{{e}}^{-\frac{2\pi }{z}}\Big )^{\delta }$ can be analytically extended to a single valued analytic function on the right half plane $\mathfrak {R}(z)>0$, the proof of the proposition is complete. $\square$

### Rademacher expansion for the Borwein coefficients

Let $n,N\in {\mathbb {N}}$. Following Rademacher [16] or [17, Eq. (117.1)], we have

**Fig. 1:**
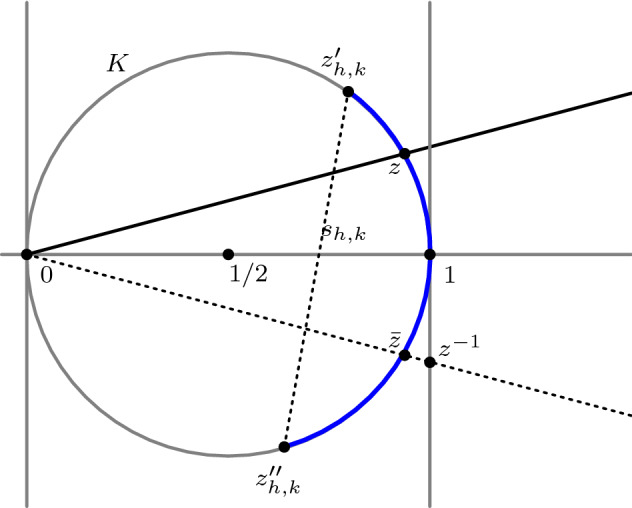
Path of integration in the *z*-plane

2.1 $$\begin{aligned} c_p^{(\delta )}(n)=\sum _{1\le k\le N}\sum _{\begin{array}{c} 0\le h<k\\ \gcd (h,k)=1 \end{array}} \frac{\mathrm i}{k^2}\mathrm{{e}}^{-\frac{2\pi \mathrm ihn}{k}}\int _{z_{h,k}'}^{z_{h,k}''} G_{p}\Big (\mathrm{{e}}^{\frac{2\pi \mathrm ih}{k}-\frac{2\pi z}{k^2}}\Big )^{\delta } \exp \bigg (\frac{2\pi nz}{k^2}\bigg )\,\mathrm dz, \end{aligned}$$

where *z* runs in each integral on an arc of the circle $K: \left| z-{1}/{2}\right| ={1}/{2}$ with $\mathfrak {R}(z)>0$, with the ends $z_{h,k}'$ and $z_{h,k}''$ (see the segment in blue in Figure 1) of the arc being given by

2.2 $$\begin{aligned} z_{h,k}'=\frac{k^2}{k^2+k_1^2}+\mathrm i\frac{kk_1}{k^2+k_1^2} \quad \text{ and }\quad z_{h,k}''=\frac{k^2}{k^2+k_2^2}-\mathrm i\frac{kk_2}{k^2+k_2^2}, \end{aligned}$$

respectively. Here $k_1,k_2\in {\mathbb {N}}$ are taken from the denominators of adjoint points of *h*/*k* in the Farey sequence of order *N*.

Applying Proposition 7 to Eq. (2.1) yields

$$\begin{aligned} c_p^{(\delta )}(n)&=\Bigg (\sum _{\begin{array}{c} 1\le k\le N\\ p\mid k \end{array}}+ \sum _{\begin{array}{c} 1\le k\le N\\ p\not \mid k \end{array}}\Bigg ) \sum _{\begin{array}{c} 0\le h<k\\ \gcd (h,k)=1 \end{array}} \frac{\mathrm i\big (\frac{p}{d}\big )^{\frac{\delta }{2}} \Big (\omega _{h,k}^{-1}\omega _{\frac{hp}{d},\frac{k}{d}}\Big )^{\delta } \mathrm{{e}}^{-\frac{2\pi \mathrm ihn}{k}}}{k^2}\\*&\quad \;\times \int _{z_{h,k}'}^{z_{h,k}''} \mathrm{{e}}^{\frac{\pi \delta (d^2-p)}{12pz}+\frac{(24n-(p-1)\delta )\pi z}{12k^2}} {\hat{G}}_{p}\Big (h,k; \mathrm{{e}}^{-\frac{2\pi }{z}}\Big )^{\delta }\,\mathrm dz\\&=:I+E. \end{aligned}$$

Notice that for the second sum *E*, the relation $p\not \mid k$ means $d=\gcd (p,k)=1$; we obtain

$$\begin{aligned} E&=\sum _{\begin{array}{c} 1\le k\le N\\ p\not \mid k \end{array}}\sum _{\begin{array}{c} 0\le h<k\\ \gcd (h,k)=1 \end{array}} \frac{\mathrm i\,p^{\frac{\delta }{2}} \Big (\omega _{h,k}^{-1}\omega _{ph,k}\Big )^{\delta } \mathrm{{e}}^{-\frac{2\pi \mathrm ihn}{k}}}{k^2}\\&\quad \times \,\int _{z_{h,k}'}^{z_{h,k}''} \mathrm{{e}}^{-\frac{\pi \delta (p-1)}{12pz}+\frac{(24n-(p-1)\delta )\pi z}{12k^2}} {\hat{G}}_{p}\Big (h,k; \mathrm{{e}}^{-\frac{2\pi }{z}}\Big )^{\delta }\,\mathrm dz. \end{aligned}$$

The path of integration in above inner sum, which is an arc of the circle *K*, can here be replaced by the chord $s_{h,k}$ from $z_{h,k}'$ to $z_{h,k}''$. On the chord $s_{h,k}$, from Rademacher [17, Eqs. (119.3) and (119.6)], we have

$$\begin{aligned} 1\le \mathfrak {R}\bigg (\frac{1}{z}\bigg ),\quad 0< \mathfrak {R}(z)<\frac{2k^2}{N^2}, \end{aligned}$$

and, from Rademacher [16, Eq. (3.5)], the length of the chord $s_{h,k}$ is

$$\begin{aligned} |s_{h,k}|<\frac{2k}{N+1}. \end{aligned}$$

Thus we deduce

$$\begin{aligned} |E|&\le \sum _{\begin{array}{c} 1\le k\le N\\ p\not \mid k \end{array}} \sum _{\begin{array}{c} 0\le h<k\\ \gcd (h,k)=1 \end{array}} \frac{ p^{\frac{\delta }{2}}|s_{h,k}|}{k^2}\sup _{z\in s_{h,k}} \bigg |\mathrm{{e}}^{-\frac{\pi \delta (p-1)}{12pz}+\frac{(24n-(p-1)\delta )\pi z}{12k^2}} {\hat{G}}_{p}\Big (h,k; \mathrm{{e}}^{-\frac{2\pi }{z}}\Big )^{\delta }\bigg |\\&\le \sum _{\begin{array}{c} 1\le k\le N\\ p\not \mid k \end{array}} \sum _{\begin{array}{c} 0\le h<k\\ \gcd (h,k)=1 \end{array}}\frac{ 2\cdot p^{\frac{\delta }{2}} \mathrm{{e}}^{-\frac{\pi \delta (p-1)}{12p}+\frac{(24n-(p-1)\delta )\pi }{6N^2}}}{k(N+1)} \sup _{z\in s_{h,k}} \left| \frac{f\bigg (\mathrm{{e}}^{\frac{2\pi \mathrm ih_1'}{k}-\frac{2\pi }{pz}}\bigg )}{f\Big (\mathrm{{e}}^{\frac{2\pi \mathrm ih'}{k}-\frac{2\pi }{z}}\Big )}\right| ^{\delta }. \end{aligned}$$

Further, it is not difficult to see that

$$\begin{aligned} \sup _{z\in s_{h,k}}\left| f\bigg (\mathrm{{e}}^{\frac{2\pi \mathrm ih_1'}{k}-\frac{2\pi }{pz}}\bigg ) f\Big (\mathrm{{e}}^{\frac{2\pi \mathrm ih'}{k}-\frac{2\pi }{z}}\Big )^{-1}\right| \le f\Big (\mathrm{{e}}^{-\frac{2\pi }{p}}\Big )f\Big (\mathrm{{e}}^{-2\pi }\Big ), \end{aligned}$$

and hence

2.3 $$\begin{aligned} |E| \le \sum _{\begin{array}{c} 1\le k\le N\\ p\not \mid k \end{array}} \sum _{\begin{array}{c} 0\le h<k\\ \gcd (h,k)=1 \end{array}}\frac{ 2\cdot p^{\frac{\delta }{2}} \mathrm{{e}}^{-\frac{\pi \delta (p-1)}{12p}+\frac{(24n-(p-1)\delta )\pi }{6N^2}}}{k(N+1)} f\Big (\mathrm{{e}}^{-\frac{2\pi }{p}}\Big )f\Big (\mathrm{{e}}^{-2\pi }\Big ). \end{aligned}$$

For the first sum *I*, the relation $p\mid k$ means $d=\gcd (p,k)=p$; we now obtain the estimate

$$\begin{aligned} I&=\sum _{\begin{array}{c} 1\le k\le N\\ p\mid k \end{array}} \sum _{\begin{array}{c} 0\le h<k\\ \gcd (h,k)=1 \end{array}} \frac{\mathrm i\Big (\omega _{h,k}^{-1}\omega _{h,\frac{k}{p}}\Big )^{\delta } \mathrm{{e}}^{-\frac{2\pi \mathrm ihn}{k}}}{k^2}\\*&\qquad \times \int _{z_{h,k}'}^{z_{h,k}''} \mathrm{{e}}^{\frac{\pi (p-1)\delta }{12z}+\frac{(24n-(p-1)\delta )\pi z}{12k^2}} \bigg (1+\Big ({\hat{G}}_{p} \Big (h,k; \mathrm{{e}}^{-\frac{2\pi }{z}}\Big )^{\delta }-1\Big )\bigg ) \,\mathrm dz\\&=:I_M+I_R, \end{aligned}$$

where the division of *I* into the two terms $I_M$ and $I_R$ comes from splitting the last factor in the integrand into 1 and $\Big ({\hat{G}}_{p}\Big (h,k; \mathrm{{e}}^{-\frac{2\pi }{z}}\Big )^{\delta }-1\Big )$. Similar to the estimate for *E*, for $I_R$ we have

$$\begin{aligned} |I_R|\le \sum _{\begin{array}{c} 1\le k\le N\\ p\mid k \end{array}}&\qquad \sum _{\begin{array}{c} 0\le h<k\\ \gcd (h,k)=1 \end{array}} \frac{2\mathrm{{e}}^{\frac{(24n-(p-1)\delta )\pi z}{6N^2}}}{k(N+1)} \sup _{z\in s_{h,k}}\bigg |\mathrm{{e}}^{\frac{\pi (p-1)\delta }{12z}} \Big ({\hat{G}}_{p}\Big (h,k; \mathrm{{e}}^{-\frac{2\pi }{z}}\Big )^{\delta }-1\Big )\bigg |. \end{aligned}$$

For all $t_1,t_2\in {{\mathbb {C}}}$ with $|t_1|, |t_2|<1$ and $\delta >0$, it not difficult to show that

$$\begin{aligned} \bigg |\Big (f(t_1)f(t_2)^{-1}\Big )^{\delta }-1\bigg | \le f(|t_1|)^{\delta }f(|t_2|)^{\delta }-1, \end{aligned}$$

by using the definition of *f*(*t*). Hence we obtain

$$\begin{aligned}&\sup _{z\in s_{h,k}} \bigg |\mathrm{{e}}^{\frac{\pi \delta (p-1)}{12pz}} \Big ({\hat{G}}_{p,\delta }\Big (h,k; \mathrm{{e}}^{-\frac{2\pi }{z}}\Big )-1\Big )\bigg |\\&\quad \le \sup _{z\in s_{h,k}}\bigg |\mathrm{{e}}^{\frac{\pi \delta (p-1)}{12}\mathfrak {R}(z^{-1})} \Big (f\Big (\mathrm{{e}}^{-2p\pi \mathfrak {R}(z^{-1})}\Big )^{\delta } f\Big (\mathrm{{e}}^{-2\pi \mathfrak {R}(z^{-1})}\Big )^{\delta }-1\Big )\bigg |\\&\quad \le \mathrm{{e}}^{\frac{\pi \delta (p-1)}{12}}\bigg (f\Big (\mathrm{{e}}^{-2p\pi }\Big )^{\delta } f\Big (\mathrm{{e}}^{-2\pi }\Big )^{\delta }-1\bigg ), \end{aligned}$$

by using $\delta \in (0,24/(p-1)]$ and the definition of ${\hat{G}}_{p}(h,k; \mathrm{{e}}^{-\frac{2\pi }{z}})$. Therefore

2.4 $$\begin{aligned} |I_R|\le \sum _{\begin{array}{c} 1\le k\le N\\ p\mid k \end{array}} \sum _{\begin{array}{c} 0\le h<k\\ \gcd (h,k)=1 \end{array}} \frac{ 2\mathrm{{e}}^{\frac{\pi \delta (p-1)}{12}+\frac{(24n-(p-1)\delta )\pi }{6N^2}}}{k(N+1)} \bigg (f\Big (\mathrm{{e}}^{-2p\pi }\Big )^{\delta } f\Big (\mathrm{{e}}^{-2\pi }\Big )^{\delta }-1\bigg ). \end{aligned}$$

To evaluate $I_M$ we split integral into two parts, $I_{MM}$ and $I_{ME}$, as indicated below; the path of integration of the first part is the whole circle *K*, traversed from 0 to 0 in negative direction, while in the second part the arc of the circle is traversed from $z_{h,k}'$ to $z_{h,k}''$ (which itself is split into a difference of two integrals with respective paths of integration along the arc starting in 0). Specifically, we have

$$\begin{aligned} I_M&=\sum _{\begin{array}{c} 1\le k\le N\\ p\mid k \end{array}} \sum _{\begin{array}{c} 0\le h<k\\ \gcd (h,k)=1 \end{array}} \frac{\mathrm i\Big (\omega _{h,k}^{-1}\omega _{h,\frac{k}{p}}\Big )^{\delta } \mathrm{{e}}^{-\frac{2\pi \mathrm ihn}{k}}}{k^2}\\*&\qquad \times \Bigg (\int _{K}-\Bigg (\int _{0}^{z_{h,k}'}-\int _{0}^{z_{h,k}''}\Bigg )\Bigg ) \mathrm{{e}}^{\frac{\pi \delta (p-1)}{12z}+\frac{(24n-(p-1)\delta )\pi z}{12k^2}}\,\mathrm dz\\&=:I_{MM}+I_{ME}. \end{aligned}$$

We estimate the second part, $I_{ME}$, first. On the arc from 0 to $z_{h,k}'$, and the arc from 0 to $z_{h,k}''$, from Rademacher [17, Eq. (119.6)] and [17, Eq. (120.2)], we have

$$\begin{aligned} 0\le \mathfrak {R}(z)\le \max (\mathfrak {R}(z_{h,k}'),\mathfrak {R}(z_{h,k}'')) <\frac{2k^2}{N^2}, \end{aligned}$$

and $\mathfrak {R}(1/z)=1$. The lengths of both, the arc from 0 to $z_{h,k}'$, and the arc from 0 to $z_{h,k}''$, are less than

$$\begin{aligned} \frac{\pi }{2}|z_{h,k}'|, \;\frac{\pi }{2}|z_{h,k}''| \le \frac{\pi k}{\sqrt{2}N}. \end{aligned}$$

Together, we obtain

2.5 $$\begin{aligned} |I_{ME}|\le 2\sum _{\begin{array}{c} 1\le k\le N\\ p\mid k \end{array}} \sum _{\begin{array}{c} 0\le h<k\\ \gcd (h,k)=1 \end{array}} \frac{1}{k^2}\frac{\pi k}{\sqrt{2}N} \mathrm{{e}}^{\frac{\pi \delta (p-1)}{12}+\frac{(24n-(p-1)\delta )\pi }{6N^2}}. \end{aligned}$$

Finally, for the first part which is

$$\begin{aligned} I_{MM}=\sum _{\begin{array}{c} 1\le k\le N\\ p\mid k \end{array}} \sum _{\begin{array}{c} 0\le h<k\\ \gcd (h,k)=1 \end{array}} \frac{\mathrm i\Big (\omega _{h,k}^{-1}\omega _{h,\frac{k}{p}}\Big )^{\delta } \mathrm{{e}}^{-\frac{2\pi \mathrm ihn}{k}}}{k^2}\int _{K} \mathrm{{e}}^{\frac{\pi \delta (p-1)}{12z}+\frac{(24n-(p-1)\delta )\pi z}{12k^2}}\,\mathrm dz, \end{aligned}$$

we use the integral representation for the modified Bessel function $I_1$ in (1.9), and apply the substitution $w=1/z$. Simplification then gives

2.6 $$\begin{aligned} I_{MM}=\frac{2\pi \delta ^{1/2}}{\sqrt{\frac{24n}{p-1}-\delta }} \sum _{\begin{array}{c} 1\le k\le N\\ p\mid k \end{array}}A_{k,\delta }(n) I_1\Bigg (\frac{(p-1)\pi }{6k} \sqrt{\delta \bigg (\frac{24n}{p-1}-\delta \bigg )}\Bigg ), \end{aligned}$$

where

$$\begin{aligned} A_{k,\delta }(n)=\frac{1}{k}\sum _{\begin{array}{c} 0\le h<k\\ \gcd (h,k)=1 \end{array}} \Big (\omega _{h,k}^{-1}\omega _{h,\frac{k}{p}}\Big )^{\delta } \mathrm{{e}}^{-\frac{2\pi \mathrm ihn}{k}}. \end{aligned}$$

From Eqs. (2.3), (2.4), (2.5) and (2.6), we therefore obtain

$$\begin{aligned}&\big |c_p^{(\delta )}(n)-I_{MM}\big |\\&\le |I_{ME}|+|I_{R}|+|E|\\&\le \frac{\mathrm{{e}}^{\frac{(p-1)\pi \delta }{12}+\frac{(24n-(p-1)\delta )\pi }{6N^2}} }{N} \sum _{\begin{array}{c} 1\le k\le N\\ p\mid k \end{array}} \frac{\varphi (k)}{k}\bigg (\pi \sqrt{2} +2\bigg (f\Big (\mathrm{{e}}^{-2p\pi }\Big )^{\delta } f\Big (\mathrm{{e}}^{-2\pi }\Big )^{\delta }-1\bigg )\bigg )\\*&\quad \;+ \sum _{\begin{array}{c} 1\le k\le N\\ 3\not \mid k \end{array}} \sum _{\begin{array}{c} 0\le h<k\\ \gcd (h,k)=1 \end{array}} \frac{ 2\cdot p^{\frac{\delta }{2}} \mathrm{{e}}^{-\frac{\pi (p-1)\delta }{12p}+\frac{(24n-(p-1)\delta )\pi }{6N^2}}}{k(N+1)} f\Big (\mathrm{{e}}^{-\frac{2\pi }{p}}\Big )^{\delta }f\Big (\mathrm{{e}}^{-2\pi }\Big )^{\delta }, \end{aligned}$$

where $\varphi (k)$ is Euler’s totient function. Notice that if $p\mid k$ then $\varphi (k)\le (1-1/p)k$. Substituting $N\mapsto pN$, the error term becomes

$$\begin{aligned}&\big |c_p^{(\delta )}(n)-I_{MM}\big |\\&\le \frac{(p-1)\mathrm{{e}}^{\frac{(p-1)\pi \delta }{12}+ \frac{(24n-(p-1)\delta )\pi }{6(pN)^2}} }{p^2} \bigg (\pi \sqrt{2}+2\bigg (f\Big (\mathrm{{e}}^{-6\pi }\Big )^{\delta } f\Big (\mathrm{{e}}^{-2\pi }\Big )^{\delta }-1\bigg )\bigg )\\*&\quad \;+ \frac{ 2(p-1)\cdot \mathrm{{e}}^{-\frac{\pi (p-1)\delta }{12p}+\frac{(24n-(p-1)\delta )\pi }{6(pN)^2}}}{p^{1-\delta /2}}f\Big (\mathrm{{e}}^{-\frac{2\pi }{p}}\Big )^{\delta } f\Big (\mathrm{{e}}^{-2\pi }\Big )^{\delta }\\&= \mathrm{{e}}^{\frac{(24n-(p-1)\delta )\pi }{6p^2N^2}} \Bigg (\frac{(p-1)\mathrm{{e}}^{\frac{(p-1)\pi \delta }{12}} }{p^2} \bigg (\pi \sqrt{2}-2+2f\Big (\mathrm{{e}}^{-6\pi }\Big )^{\delta } f\Big (\mathrm{{e}}^{-2\pi }\Big )^{\delta }\bigg )\\*&\quad \; + \frac{ 2(p-1)\cdot \mathrm{{e}}^{-\frac{\pi (p-1)\delta }{12p}}}{p^{1-\delta /2}} f\Big (\mathrm{{e}}^{-\frac{2\pi }{p}}\Big )^{\delta } f\Big (\mathrm{{e}}^{-2\pi }\Big )^{\delta }\Bigg ). \end{aligned}$$

This completes the proof of Theorem 3. $\square$

## Sign pattern for the Borwein coefficients with $p=3$

### The proof of Theorem 4

We now assume that $\delta \in (0, 3]$, and start with $n\ge 1$ and will later restrict to $n\ge 158$. We let

3.1 $$\begin{aligned} L_{\delta ,n}=\frac{\pi }{18}\sqrt{\delta (12n-\delta )},\quad {\hat{c}}_{\delta }(n)=\frac{2\pi ^2\delta }{27} L_{\delta ,n}^{-1}\, I_1\big (2L_{\delta ,n}\big ) \end{aligned}$$

(remember that $I_1$ is the modified Bessel function, defined in (1.8)), and fix

$$\begin{aligned} N= \Big \lceil \big (20 L_{\delta ,n}^2/\delta \big )^{1/2}\Big \rceil . \end{aligned}$$

Substituting these into Theorem 3 and taking $p=3$, using computer algebra (we utilized *Mathematica* and found it convenient to rewrite the occurrences of $f(\cdot )$ using $f(\mathrm{{e}}^{-2\pi x})=\mathrm{{e}}^{2\pi x/24}\eta (\mathrm ix)^{-1}$ where $\eta (x)$ is the classical Dedekind eta function, already implemented as a built-in function in *Mathematica*), we find that

3.2 $$\begin{aligned} \Big |E_{3,N}^{(\delta )}(n)\Big |\le \frac{4}{9}\cdot \mathrm{{e}}^{\frac{3}{5\pi }} \Big (1.689^{\delta }\big (1.222+1.002^{\delta }\big )+3\cdot 1.692^{\delta }\Big ) \end{aligned}$$

for any integer $n\ge 1$. Furthermore, by noticing that $\varphi (3k)\le 2k$, we have

3.3 $$\begin{aligned}&\bigg |c_{\delta }(n)-{\hat{c}}_{\delta }(n) \cos \bigg (\frac{\pi \delta }{18}+\frac{2\pi n}{3}\bigg )\bigg |\nonumber \\&\qquad \qquad \le \Big |E_{3,N}^{(\delta )}(n)\Big |+ \frac{2\pi \delta ^{1/2}}{\sqrt{12n-\delta }} \sum _{2\le k\le N}\frac{\varphi (3k)}{3k}\, I_1\Big (\frac{\pi }{9k}\sqrt{\delta (12n-\delta )}\Big )\nonumber \\&\qquad \qquad \le \Big |E_{3,N}^{(\delta )}(n)\Big |+ \frac{2\pi ^2\delta }{27L_{\delta , n}} \sum _{2\le k\le N}I_1\bigg (\frac{2}{k}L_{\delta ,n}\bigg ). \end{aligned}$$

To give an upper bound for the above sum we require the following lemma.

#### Lemma 8

For any real $x>0$ and integer $y>2$ we have

$$\begin{aligned} \sum _{2\le k\le y}I_1\bigg (\frac{2x}{k}\bigg )\le x\log y+2I_1\left( x\right) -\bigg (2-\gamma -\frac{1}{2y}\bigg )x. \end{aligned}$$

Here $\gamma =0.577216\ldots$ is the Euler–Mascheroni constant.

#### Proof

Using the well-known bound

$$\begin{aligned} \sum _{1\le k\le y}\frac{1}{k}\le \log y+\gamma +\frac{1}{2y}, \end{aligned}$$

we find that

$$\begin{aligned} \sum _{2\le k\le y}I_1\left( \frac{2x}{k}\right)&=\sum _{n\ge 0}\frac{x^{2n+1}}{n!(n+1)!}\sum _{2\le k\le y}\frac{1}{k^{2n+1}}\\&=x\sum _{2\le k\le y}\frac{1}{k}+ \sum _{n\ge 1}\frac{x^{2n+1}}{n!(n+1)!}\sum _{2\le k\le y}\frac{1}{k^{2n+1}}\\&\le x\bigg (\log y+\gamma -1+\frac{1}{2y}\bigg )+ 2\Bigg (\sum _{n\ge 0}\frac{(x/2)^{2n+1}}{n!(n+1)!}-\frac{x}{2}\Bigg )\\&\le x\log y+2I_1\left( x\right) -\bigg (2-\gamma -\frac{1}{2y}\bigg )x, \end{aligned}$$

which completes the proof. $\square$

Now, applying Lemma 8 to (3.3), we have

$$\begin{aligned}&\bigg |c_{\delta }(n)-{\hat{c}}_{\delta }(n) \cos \bigg (\frac{\pi \delta }{18} +\frac{2\pi n}{3}\bigg )\bigg |\\&\qquad \le \,\Big |E_{3,N}^{(\delta )}(n)\Big |+\frac{2\pi ^2\delta }{27L_{\delta , n}} \big (L_{\delta ,n}\log N+2I_1(L_{\delta ,n}) -(2-\gamma -(2N)^{-1})L_{\delta ,n}\big ). \end{aligned}$$

Now we let $n\ge 158$. Since

$$\begin{aligned} 20 L_{\delta ,n}^2/\delta =\frac{20\pi ^2(12n-\delta )}{18^2}\ge \frac{20\pi ^2\cdot (12\cdot 158-3)}{18^2}\ge 1153, \end{aligned}$$

we have

$$\begin{aligned} N=\Big \lceil (20 L_{\delta ,n}^2/\delta )^{1/2}\Big \rceil \le \big (1+1153^{-1/2}\big )\big (20 L_{\delta ,n}^2/\delta \big )^{1/2}. \end{aligned}$$

If we also insert the definition of ${\hat{c}}_{\delta }(n)$ we obtain that

$$\begin{aligned}&\bigg |\frac{c_{\delta }(n)}{{\hat{c}}_{\delta }(n)}- \cos \bigg (\frac{\pi \delta }{18}+\frac{2\pi n}{3}\bigg )\bigg |\\&\le \frac{L_{\delta ,n}\log L_{\delta ,n}+2 I_1(L_{\delta ,n})}{I_1(2L_{\delta ,n})}\\&\quad \;+\frac{\Big |E_{3,N}^{(\delta )}(n)\Big |+\frac{2\pi ^2\delta }{27} \Big (\log \big (\big (1+1153^{-1/2}\big )(20/\delta )^{1/2}\big ) -\big (2-\gamma -1153^{-1/2}/2\big )\Big )}{\frac{2\pi ^2\delta }{27} L_{\delta ,n}^{-1}\,I_1(2L_{\delta ,n})}. \end{aligned}$$

Using *Mathematica* and inserting (3.2), we obtain

$$\begin{aligned} \left| \frac{c_{\delta }(n)}{{\hat{c}}_{\delta }(n)}- \cos \left( \frac{\pi \delta }{18}+\frac{2\pi n}{3}\right) \right| \le \frac{L_{\delta ,n} w(\delta )+L_{\delta ,n}\log L_{\delta ,n}+ 2 I_1(L_{\delta ,n})}{I_1\left( 2L_{\delta ,n}\right) }, \end{aligned}$$

with

3.4 $$\begin{aligned} w(\delta )=\frac{1}{2}\log \bigg (\frac{1}{\delta }\bigg )+ \frac{0.736\,\big (1.689^{\delta }\big (1.222+1.002^{\delta }\big )+ 3\cdot 1.692^{\delta }\big )}{\delta }+0.119. \end{aligned}$$

This completes the proof of Theorem 4. $\square$

### The proof of Corollary 5

To establish Corollary 5, we will make use of the following lemma.

#### Lemma 9

Let $L_{\delta ,n}$ and $w(\delta )$ be given as in (3.1) and (3.4), respectively. For each $\delta \in (0, 3]$, define

$$\begin{aligned} M(L_{\delta ,n}):=\frac{L_{\delta ,n} w(\delta )+L_{\delta ,n}\log L_{\delta ,n} +2 I_1(L_{\delta ,n})}{I_1\left( 2L_{\delta ,n}\right) }. \end{aligned}$$

Then $M(L_{\delta ,n})$ is decreasing in *n* for $n\ge 158$, whenever $\delta \in [0.227, 3]$.

#### Proof

We have $L_{\delta ,n}=(\pi /18)\sqrt{\delta (12n-\delta )}\ge 3.6$ for all $\delta \in [0.227,3]$ and $n\ge 158$, and $L_{\delta ,n}$ is increasing for all $n\ge 1$. Hence we just need to prove that

$$\begin{aligned} M(u):=\frac{u w(\delta )+u\log u+2 I_1(u)}{I_1\left( 2u\right) }, \end{aligned}$$

is decreasing for all $u\ge 3$. We have $w(\delta )>0$ for all $\delta \in [0.227, 3]$. Using the definition of $I_1(u)$ in (1.8), it is clear that $uw(\delta )/I_1(2u)$ is decreasing for $u>0$. Also,

$$\begin{aligned} \bigg (\frac{u\log u}{I_1(2u)}\bigg )'= \frac{u^{-1}(u^{-1}I_1(2u))-(u^{-1}I_1(2u))'\log u}{(u^{-1}I_1(2u))^2}, \end{aligned}$$

and

$$\begin{aligned} u^{-1}(u^{-1}I_1(2u))-(u^{-1}I_1(2u))'\log u&=\sum _{\ell \ge 0}\frac{u^{2\ell -1}}{\ell !(\ell +1)!}- \log u \sum _{\ell \ge 0}\frac{2\ell u^{2\ell -1}}{\ell !(\ell +1)!}\\&=\frac{1}{u}- \sum _{\ell \ge 1}\frac{(2\ell \log u-1) u^{2\ell -1}}{\ell !(\ell +1)!}\\&\le \frac{1}{u}-\frac{u}{2}(2\log u-1), \end{aligned}$$

for all $u\ge \sqrt{e}$. Clearly, $1/u-u(2\log u-1)/2$ is decreasing and not greater than

$$\begin{aligned} \frac{1}{3}-\frac{3}{2}(2\log 3-1)<0, \end{aligned}$$

for $u\ge 3$. This means that ${u\log u}/{I_1(2u)}$ is decreasing for $u\ge 3$.

The more difficult part is to prove that $I_1(u)/I_1(2u)$ is decreasing for $u\ge 3$. We shall prove

3.5 $$\begin{aligned} \bigg (\frac{I_1(u)}{I_1(2u)}\bigg )' =\frac{I_1'(u)I_1(2u)-2I_1(u)I_1'(2u)}{I_1(2u)^2}\le 0, \end{aligned}$$

for all $u>0$. Inserting the well-known functional relation for the modified Bessel function $I_1$, namely $I_1'(u)=I_0(u)-u^{-1}I_1(u)$, into the above equation, we obtain

$$\begin{aligned} I_1'(u)I_1(2u)-2I_1(u)I_1'(2u)&=I_0(u)I_1(2u)-2I_0(2u)I_1(u)\\&=\frac{1}{u}\bigg (\frac{uI_0(u)}{I_{1}(u)}-\frac{2uI_0(2u)}{I_{1}(2u)}\bigg ). \end{aligned}$$

By using a result of Simpson and Spector [21] on the monotonicity of the ratios of modified Bessel functions $uI_v(u)/I_{v+1}(u), (v\ge 0)$, namely, that for all $v\ge 0$, $uI_v(u)/I_{v+1}(u)$ is strictly monotone decreasing on $(0, \infty )$, we arrive at the inequality in (3.5). $\square$

We shall prove for all $n\ge 158$ that

$$\begin{aligned} \bigg |\cos \bigg (\frac{\pi \delta }{18}+\frac{2\pi n}{3}\bigg )\bigg |> M(L_{\delta , n}). \end{aligned}$$

From this and Theorem 4 we see that $c_{\delta }(n)$ has the same sign as $\cos \Big (\frac{\pi \delta }{18}+\frac{2\pi n}{3}\Big )$, and hence the proof of Corollary 5 follows. This is because $\cos (\pi \delta /18+2\pi n/3)$ is a periodic function in *n* of period 3, and because of Lemma 9 above. We just need to prove that

$$\begin{aligned} \bigg |\cos \bigg (\frac{\pi \delta }{18}+\frac{2\pi j}{3}\bigg )\bigg |> M(L_{\delta , 158}) \end{aligned}$$

holds for all $j\in \{0,1,2\}$ and $\delta \in [0.227, 2.9999]$. This can be verified by *Mathematica*.

## Arithmetic properties of the cubic Borwein coefficients

For convenience, we use standard *q*-series notation (cf. [10]). For $a\in {{\mathbb {C}}}$ and $0<|q|<1$, let

$$\begin{aligned} (a;q)_\infty :=\prod _{j=0}^\infty (1-a q^j), \end{aligned}$$

and

$$\begin{aligned} (a_1,\ldots ,a_m;q)_\infty :=(a_1;q)_\infty \cdots (a_m;q)_\infty . \end{aligned}$$

Further let the modified Jacobi theta function be defined by

$$\begin{aligned} \theta (z;q):=(z,q/z;q)_\infty =\frac{1}{(q;q)_\infty }\sum _{n\in {\mathbb {Z}}} (-1)^n q^{\frac{n(n-1)}{2}}z^n, \end{aligned}$$

where the last equation is equivalent to Jacobi’s triple product identity [10, Eq. (1.6.1)].

In this section we are interested in arithmetic properties (including sign patterns, vanishing and divisibility properties) of the Borwein coefficients for exponent $\delta =3$, i.e. for

$$\begin{aligned} c_k^{(3)}(n):=\big [q^n\big ]\frac{(q;q)_\infty ^3}{(q^k;q^k)_\infty ^3} \end{aligned}$$

where $k>1$ is an integer, which we shall refer to as *cubic* Borwein coefficients. (Here we relax the condition that *k* is a prime, which we assumed in the earlier sections. Nevertheless, in relevant cases, *k* will be assumed to be odd, or even a prime.)

We deduce the arithmetic properties we are interested in from the following result.

### Theorem 10

If *k* is a positive even integer then

$$\begin{aligned} (q;q)_{\infty }^3&= \sum _{0\le \ell < \frac{k-1}{2}}(-1)^{\ell }q^{\frac{\ell (\ell +1)}{2}} \left( -q^{\frac{k(k-1-2\ell )}{2}},-q^{\frac{k(k+1+2\ell )}{2}},q^{k^2}; q^{k^2}\right) _{\infty }\\*&\quad \;\times \left( 2\ell +1-2k\sum _{n\ge 0} \left( \frac{q^{k(kn+\frac{k-1-2\ell }{2})}}{1+q^{k(kn+\frac{k-1-2\ell }{2})}}- \frac{q^{k(kn+\frac{k+1+2\ell }{2})}}{1+q^{k(kn+\frac{k+1+2\ell }{2})}}\right) \right) . \end{aligned}$$

If *k* is a positive odd integer then

$$\begin{aligned} (q;q)_{\infty }^3&- (-1)^{\frac{k-1}{2}}kq^{\frac{k^2-1}{8}}(q^{k^2};q^{k^2})_{\infty }^3\\&=\sum _{0\le \ell < \frac{k-1}{2}}(-1)^{\ell }q^{\frac{\ell (\ell +1)}{2}} \left( q^{\frac{k(k-1-2\ell )}{2}},q^{\frac{k(k+1+2\ell )}{2}},q^{k^2}; q^{k^2}\right) _{\infty }\\*&\quad \;\times \left( 2\ell +1+2k\sum _{n\ge 0} \left( \frac{q^{k(kn+\frac{k-1-2\ell }{2})}}{1-q^{k(kn+\frac{k-1-2\ell }{2})}}- \frac{q^{k(kn+\frac{k+1+2\ell }{2})}}{1-q^{k(kn+\frac{k+1+2\ell }{2})}}\right) \right) . \end{aligned}$$

Notice that the right-hand sides of the two identities above are finite sums whose terms involve Jacobi triple products and Lambert series. The following corollaries are direct consequences of Theorem 10 whose proof we give at the end of this section.

Choosing $k=2$ in Theorem 10, we have

$$\begin{aligned} (q;q)_{\infty }^3=\left( -q,-q^3,q^{4};q^{4}\right) _{\infty }\left( 1-4\sum _{n\ge 0} \left( \frac{q^{4n+1}}{1+q^{4n+1}}-\frac{q^{4n+3}}{1+q^{4n+3}}\right) \right) . \end{aligned}$$

Replacing *q* by $-q$, we get

$$\begin{aligned} \frac{(-q;-q)_{\infty }^3}{\left( q,q^3,q^{4};q^{4}\right) _{\infty }} =1+4\sum _{n\ge 0}\left( \frac{q^{4n+1}}{1-q^{4n+1}}- \frac{q^{4n+3}}{1-q^{4n+3}}\right) . \end{aligned}$$

After simplification and an application of Jacobi’s triple product identity we obtain the following classical result (cf. [3, Eq. (3.2.8)]).

### Corollary 11

(Sum of two squares theorem) For $k=2$ we have

$$\begin{aligned} \bigg (\sum _{n=-\infty }^\infty q^{n^2}\bigg )^2 =(-q,-q,q^2;q^2)_{\infty }^2= 1+4\sum _{n\ge 0}\bigg (\frac{q^{4n+1}}{1-q^{4n+1}}- \frac{q^{4n+3}}{1-q^{4n+3}}\bigg ). \end{aligned}$$

Similarly, choosing $k=3$ in Theorem 10, we readily obtain the following result which can be interpreted as an identity for the cubic theta functions of the Borwein brothers [5].

### Corollary 12

(A cubic theta function addition formula) For $k=3$ we have

4.1 $$\begin{aligned} \frac{(q,q)_{\infty }^3+3q(q^9;q^9)_{\infty }^3}{(q^3;q^3)_{\infty }}= 1+6\sum _{n\ge 0}\bigg (\frac{q^{9n+3}}{1-q^{9n+3}}- \frac{q^{9n+6}}{1-q^{9n+6}}\bigg ). \end{aligned}$$

The connection of Corollary 12 to the cubic theta functions is as follows: For

$$\begin{aligned} L(q):=\sum _{n,m=-\infty }^\infty q^{n^2+nm+m^2} \end{aligned}$$

the Borweins, in [5, p. 695] defined the following three cubic analogues of Jacobi theta functions,

4.2 $$\begin{aligned} a(q):=L(q),\quad \; b(q):=\big [3L(q)^3-L(q)\big ]/2,\quad \; c(q):=\big [L(q^{1/3}-L(q)\big ]/2. \end{aligned}$$

(Explicit series representations for *a*(*q*), *b*(*q*) and *c*(*q*) are conveniently listed in [6, Eqs. (1.6)–(1.8)].) Now the Lambert series for *L*(*q*) (thus for *a*(*q*)) is

4.3 $$\begin{aligned} L(q)=1+6\sum _{n\ge 0}\bigg (\frac{q^{3n+1}}{1-q^{3n+1}}- \frac{q^{3n+2}}{1-q^{3n+2}}\bigg ), \end{aligned}$$

which is originally due to Lorenz [15, p. 11]. See [6, p. 43] for a discussion on the history of (4.3) including alternative proofs. While a central result in the theory of Borweins’ cubic theta functions is the cubic identity [5, Eq. (2.3)]

$$\begin{aligned} a(q)^3=b(q)^3+c(q)^3, \end{aligned}$$

many other identities that connect the three cubic theta functions *a*(*q*), *b*(*q*), *c*(*q*) exist in addition, including (cf. [9, Eq. (3.30)])

4.4 $$\begin{aligned} a(q^3)=b(q)+c(q^3), \end{aligned}$$

which is immediate from the defining relations (4.2). Now since (cf. [6, Proposition 2.2])

4.5 $$\begin{aligned} b(q)=\frac{(q;q)_\infty ^3}{(q^3;q^3)_\infty },\quad \text {and}\quad c(q)=3q^{\frac{1}{3}}\frac{(q^3;q^3)_\infty ^3}{(q;q)_\infty }, \end{aligned}$$

it is clear that (4.1) is nothing else than (4.4) in explicit terms.

In the case that *k* is an odd positive integer, the Lambert series appearing in the statement of Theorem 10 contain only non-zero coefficients of powers of *q* whose exponents are multiples of *k*. The triple product however has the prefactor $q^{\ell (\ell +1)/2}$ which is of relevance. Since $\ell (\ell +1)/2\equiv h\pmod k$ is equivalent to $(2\ell +1)^2\equiv 1+8h\pmod k$, the following corollary is immediate.

### Corollary 13

Let *k* be an odd positive integer and *h* be a non-negative integer less than *k* such that

$$\begin{aligned} \big |\big \{\ell \!\!\!\pmod k: (2\ell +1)^2\equiv 1+8h\!\!\!\pmod k\big \}\big |=0. \end{aligned}$$

Then

$$\begin{aligned} c_{k}^{(3)}(kn+h)=0,\qquad \text {for all}~n\in {\mathbb {N}}_0. \end{aligned}$$

In particular, for the cases $k=3,5,7,9$, we have

$$\begin{aligned} c_3^{(3)}(3n+2)&=0,\\ c_5^{(3)}(5n+2)&=c_5^{(3)}(5n+4)=0,\\ c_7^{(3)}(7n+2)&=c_7^{(3)}(7n+4)=c_7^{(3)}(7n+5)=0,\\ c_9^{(3)}(9n+2)&=c_9^{(3)}(9n+4)=c_9^{(3)}(9n+5)=c_9^{(3)}(9n+7) =c_9^{(3)}(9n+8)=0, \end{aligned}$$

for all $$n\in {\mathbb {N}}_0$$.

We now show that for any odd prime *p* the cubic Borwein coefficients have a sign pattern of period *p*, as stated in Theorem 6.

### Proof of Theorem 6

Notice that if *p* is an odd prime and $0\le \ell _1<\ell _2< \frac{p-1}{2}$ then

$$\begin{aligned} \frac{\ell _1(\ell _1+1)}{2}\not \equiv \frac{\ell _2(\ell _2+1)}{2}\pmod p, \end{aligned}$$

and for all $0\le \ell < \frac{p-1}{2}$ the expression

$$\begin{aligned} \frac{(q^p,q^{\ell },q^{p-\ell };q^p)_{\infty }}{(q;q)_{\infty }^3}&\sum _{n\ge 0}\left( \frac{q^{pn+\ell }}{1-q^{pn+\ell }} -\frac{q^{pn+p-\ell }}{1-q^{pn+p-\ell }}\right) \\&=\frac{(q^p,q^{\ell },q^{p-\ell };q^p)_{\infty }}{(q;q)_{\infty }^3}\sum _{n\ge 0}\frac{q^{pn+\ell }(1-q^{p-2\ell })}{(1-q^{pn+\ell })(1-q^{pn+p-\ell })}\\&=\frac{(q^{\ell },q^{p-\ell };q^p)_{\infty }}{(q;q)_{\infty }^2} \sum _{n\ge 0}\frac{q^{pn+\ell }(1-q^{p-2\ell })}{(1-q^{pn+\ell })(1-q^{pn+p-\ell })}\\&\quad \times \,\prod _{\begin{array}{c} n\ge 1\\ n\not \equiv 0\pmod p \end{array}}\frac{1}{1-q^n} \end{aligned}$$

is in ${\mathbb {N}}[[q]]$, i.e., a power series in *q* with positive coefficients. Replacing *q* by $q^p$ and $\ell$ by $(p-1-2\ell )/2$, the above expression is in ${\mathbb {N}}[[q^p]]$. Also notice that for all odd positive integers *k*, and all integers $0\le \ell <\frac{k-1}{2}$,

$$\begin{aligned} \frac{k^2-1}{8}-\frac{\ell (\ell +1)}{2}\not \equiv 0\pmod k. \end{aligned}$$

Theorem 6 now readily follows from Theorem 10. $\square$

As a by-product of the above proof, due to the appearance of the factor $kq^{\frac{k^2-1}{8}}$ (which trivially is divisible by *k*) in the second formula in Theorem 10, we have the following result:

### Corollary 14

Let *k* be an odd positive integer. Then

$$\begin{aligned} c_k^{(3)}\bigg (kn+\frac{k^2-1}{8}\bigg )\equiv 0\pmod k, \qquad \text {for all } n\in {\mathbb {N}}_0. \end{aligned}$$

Before we prove Theorem 10, we give a proposition and a lemma.

### Proposition 15

For each $k\in {\mathbb {N}}$ we have

$$\begin{aligned} \theta (z;q)=\frac{\big (q^{k^2};q^{k^2}\big )_{\infty }}{(q;q)_{\infty }} \sum _{\left\lceil \frac{1-k}{2}\right\rceil \le \ell \le \left\lceil \frac{k-1}{2}\right\rceil } (-1)^{\ell }q^{\frac{\ell (\ell -1)}{2}}z^{\ell } \theta \Big ((-1)^{k-1}z^{k}q^{\frac{k(k-1+2\ell )}{2}};q^{k^2}\Big ). \end{aligned}$$

### Proof

By Jacobi’s triple product identity and dissection of the sum into residue classes modulo *k*, we have

$$\begin{aligned} \theta (z;q)&=\frac{1}{(q;q)_{\infty }} \sum _{\left\lceil \frac{1-k}{2}\right\rceil \le \ell \le \left\lceil \frac{k-1}{2}\right\rceil } (-1)^{\ell }q^{\frac{\ell (\ell -1)}{2}}z^{\ell } \sum _{n\in {\mathbb {Z}}}(-1)^{kn} q^{k^2\frac{n(n-1)}{2}+\frac{nk(k-1)}{2}+kn\ell }z^{kn}\\&=\frac{\big (q^{k^2};q^{k^2}\big )_{\infty }}{(q;q)_{\infty }}\sum _{\left\lceil \frac{1-k}{2}\right\rceil \le \ell \le \left\lceil \frac{k-1}{2}\right\rceil } (-1)^{\ell }q^{\frac{\ell (\ell -1)}{2}}z^{\ell } \theta \Big ((-1)^{k-1}z^{k}q^{\frac{k(k-1+2\ell )}{2}};q^{k^2}\Big ), \end{aligned}$$

which completes the proof. $\square$

### Lemma 16

Let $$k\in {\mathbb {Z}}$$ and $$\alpha \in (0,1)$$.

$$\begin{aligned}&\frac{\,\mathrm d}{\,\mathrm dx}\bigg |_{x=0}\theta \big ((-1)^kq^{\alpha }\mathrm{{e}}^{-x};q\big )\\&\qquad =\,\theta \big ((-1)^kq^{\alpha };q\big ) \sum _{n\ge 0}\bigg (\frac{(-1)^kq^{n+\alpha }}{1-(-1)^kq^{n+\alpha }} -\frac{(-1)^kq^{n+1-\alpha }}{1-(-1)^kq^{n+1-\alpha }}\bigg ). \end{aligned}$$

### Proof

We compute

$$\begin{aligned}&\frac{\,\mathrm d}{\,\mathrm dx}\bigg |_{x=0}\frac{\theta \big ((-1)^kq^{\alpha }\mathrm{{e}}^{-x};q\big )}{\theta \big ((-1)^kq^{\alpha };q\big )}\\&\quad =\frac{\,\mathrm d}{\,\mathrm dx}\bigg |_{x=0}\log \theta \big ((-1)^kq^{\alpha }\mathrm{{e}}^{-x};q\big )\\&\quad =\sum _{n\ge 0}\frac{\,\mathrm d}{\,\mathrm dx}\bigg |_{x=0}\log \Big (\big (1-(-1)^kq^{n+\alpha }\mathrm{{e}}^{-x}\big ) \big (1-(-1)^kq^{n+1-\alpha }\mathrm{{e}}^{x}\big )\Big )\\&\quad =\sum _{n\ge 0}\bigg (\frac{(-1)^kq^{n+\alpha }}{1-(-1)^kq^{n+\alpha }}-\frac{(-1)^kq^{n+1-\alpha }}{1-(-1)^kq^{n+1-\alpha }}\bigg ), \end{aligned}$$

which completes the proof. $\square$

For $k=0$ and $\alpha \rightarrow 1^-$ we get from Lemma 16

4.6 $$\begin{aligned} \frac{\,\mathrm d}{\,\mathrm dx}\bigg |_{x=0}\theta \big (\mathrm{{e}}^{-x};q\big )= (q;q)_\infty ^2. \end{aligned}$$

After these preparations, we are ready for the proof of Theorem 10. For convenience, for a statement *A* we use the notation

$$\begin{aligned} \mathbf{1}_A={\left\{ \begin{array}{ll}1&{}\text {if}\,A\,\text {is}\,\text {true,}\\ 0&{}\text {otherwise.}\end{array}\right. } \end{aligned}$$

### Proof of Theorem 10

Using Eq. (4.6), Proposition 15 and Lemma 16, we have

$$\begin{aligned}&(q;q)_{\infty }^2=\frac{\,\mathrm d}{\,\mathrm dx}\bigg |_{x=0}\theta \big (\mathrm{{e}}^{-x};q\big )\\&\quad =\frac{\big (q^{k^2};q^{k^2}\big )_{\infty }}{(q;q)_{\infty }}\frac{\,\mathrm d}{\,\mathrm dx} \bigg |_{x=0} \sum _{\left\lceil \frac{1-k}{2}\right\rceil \le \ell \le \left\lceil \frac{k-1}{2}\right\rceil }\\&\qquad \times \,(-1)^{\ell }q^{\frac{\ell (\ell -1)}{2}}\mathrm{{e}}^{-\ell x } \theta \Big ((-1)^{k-1}\mathrm{{e}}^{-kx}q^{\frac{k(k-1+2\ell )}{2}};q^{k^2}\Big )\\&\quad =\frac{\big (q^{k^2};q^{k^2}\big )_{\infty }}{(q;q)_{\infty }} \frac{\,\mathrm d}{\,\mathrm dx}\bigg |_{x=0} \sum _{\frac{1-k}{2}<\ell \le \left\lceil \frac{k-1}{2}\right\rceil }\\&\qquad \times \,(-1)^{\ell }q^{\frac{\ell (\ell -1)}{2}}\mathrm{{e}}^{-\ell x } \theta \Big ((-1)^{k-1}\mathrm{{e}}^{-kx}q^{\frac{k(k-1+2\ell )}{2}};q^{k^2}\Big )\\*&\qquad +\,\mathbf{1}_{k\equiv 1\!\!\!\!\pmod 2} \frac{\big (q^{k^2};q^{k^2}\big )_{\infty }}{(q;q)_{\infty }}\frac{\,\mathrm d}{\,\mathrm dx}\bigg |_{x=0}(-1)^{\frac{1-k}{2}} q^{\frac{k^2-1}{8}}\mathrm{{e}}^{-\frac{1-k}{2} x } \theta \Big ((-1)^{k-1}\mathrm{{e}}^{-kx};q^{k^2}\Big )\\&\quad =\frac{\big (q^{k^2};q^{k^2}\big )_{\infty }}{(q;q)_{\infty }} \sum _{\frac{1-k}{2}< \ell \le \left\lceil \frac{k-1}{2}\right\rceil } (-1)^{\ell }q^{\frac{\ell (\ell -1)}{2}} \theta \Big ((-1)^{k-1}q^{\frac{k(k-1+2\ell )}{2}};q^{k^2}\Big )\\&\qquad \times \,\Bigg (-\ell -k\sum _{n\ge 0} \Bigg (\frac{(-1)^kq^{k^2(n+\frac{k-1+2\ell }{2k})}}{1+(-1)^kq^{k^2(n+\frac{k-1+2\ell }{2k})}}- \frac{(-1)^kq^{k^2(n+\frac{k+1-2\ell }{2k})}}{1+(-1)^kq^{k^2(n+\frac{k+1-2\ell }{2k})}}\Bigg )\Bigg )\\*&\qquad +\,\mathbf{1}_{k\equiv 1\!\!\!\!\pmod 2}\,k \frac{\big (q^{k^2};q^{k^2}\big )_{\infty }}{(q;q)_{\infty }}(-1)^{\frac{1-k}{2}}q^{\frac{k^2-1}{8}} \big (q^{k^2};q^{k^2}\big )_\infty ^2. \end{aligned}$$

Therefore,

$$\begin{aligned}&(q;q)_{\infty }^3-(-1)^{\frac{k-1}{2}}kq^{\frac{k^2-1}{8}} \big (q^{k^2};q^{k^2}\big )_{\infty }^3\,\mathbf{1}_{k\equiv 1\!\!\!\!\pmod 2}\\&=\sum _{0\le \ell < \frac{k-1}{2}}(-1)^{\ell }q^{\frac{\ell (\ell +1)}{2}} \Big ((-1)^{k-1}q^{\frac{k(k-1-2\ell )}{2}}, (-1)^{k-1}q^{\frac{k(k+1+2\ell )}{2}},q^{k^2};q^{k^2}\Big )_{\infty }\\*&\quad \;\times \Bigg (2\ell +1-2k\sum _{n\ge 0}\Bigg (\frac{(-1)^{k} q^{k(kn+\frac{k-1-2\ell }{2})}}{1+(-1)^kq^{k(kn+\frac{k-1-2\ell }{2})}}- \frac{(-1)^{k}q^{k(kn+\frac{k+1+2\ell }{2})}}{1+(-1)^kq^{k(kn+\frac{k+1+2\ell }{2})}}\Bigg )\Bigg ). \end{aligned}$$

This completes the proof. $\square$

## Appendix 1: Further conjectures on precise sign patterns

Conjecture 2 is a statement about the *precise* (not asymptotic) sign pattern of a *q*-series with base $q^3$. We now present similar conjectures about precise sign patterns for other bases $q^m$, where *m* is a small positive integer (we choose to list the cases $m\le 12$ here). To keep the exposition short, we refrain from giving all the details about justifying the specific ranges of the exponents $\delta$ for which the respective sign patterns appear to hold. The analysis would be similar to that for Conjecture 2. We nevertheless give some hints about how the various irrational constants emerge.

We have made similar observations for products involving other bases ($q^m$ with selected $m>12$). While it should be possible to approach the conjectures asymptotically by the methods we used in this paper to treat the $m=3$ case, we believe it to be a challenge to prove the precise results (that depend on the respective specified ranges), at least in the cases $m\ne 2,6$.

### A.1 Base ${\varvec{q}}^{\varvec{2}}$

The *q*-series coefficients of the infinite Borwein product

$$\begin{aligned} G_2(q)=(q;q^2)_\infty , \end{aligned}$$

evidently have the sign pattern $+-$.

We believe that even more is true:

#### Conjecture 17

The *q*-series coefficients of $G_2(q)^\delta$ exhibit the sign pattern $+-$ for any $\delta \ge 1$.

Since

$$\begin{aligned} G_2(q)^\delta =1-\delta q+\frac{\delta (\delta -1)}{2} q^2+O(q^3), \end{aligned}$$

it is clear that if $0<\delta <1$ the coefficient of $q^2$ would be negative, in violation with the sign pattern $+-$. Further, since $G_2(-q)=(-q;q^2)_\infty$, it is clear that Conjecture 17 is true for all positive integers $\delta$. We speculate that a proof of Conjecture 17 for $\delta \in {\mathbb {R}}^+\setminus {\mathbb {N}}$ without using asymptotic machinery is feasible (yet we have none available as for now). A similar situation arises in the base $q^6$ case, see Subappendix 1 below.

### A.2 Base ${\varvec{q}}^{\varvec{5}}$

The infinite product

$$\begin{aligned} Q_5(q)=\frac{(q,q^4;q^5)_\infty }{(q^2,q^3;q^5)_\infty } \end{aligned}$$

is the well-known product for the Rogers–Ramanujan continued fraction. It was shown by Richmond and Szekeres [19] that the coefficients of $Q_5(q)$ have the sign pattern $+-+--$.

We conjecture that more is true:

#### Conjecture 18

The *q*-series coefficients of $Q_5(q)^\delta$ exhibit the sign pattern $+-+--$ for

$$\begin{aligned} 1\le \delta \le \frac{\sqrt{97}-5}{2}\approx 2.424428900898\ldots . \end{aligned}$$

For

$$\begin{aligned} 2.571366313289\ldots \approx \alpha \le \delta \le 4, \end{aligned}$$

they exhibit the sign pattern $+-+-+$. (Here $\alpha$ is the unique real root of the polynomial

$$\begin{aligned} x^7+35x^6+7x^5-6055 x^4-14336 x^3+104300 x^2-184752 x+282240 \end{aligned}$$

that satisfies $2<\alpha <3$.) For $\delta =-1$ they exhibit the sign pattern $++---$, and for $-3\le \delta \le -2$ the sign pattern $+++--$.

The constant $\frac{\sqrt{97}-5}{2}$ comes from the coefficient of $q^{4}$ in $Q_5(q)^\delta$, which contains $\delta ^2+5\delta -18$ as a factor (of which $\frac{\sqrt{97}-5}{2}$ is a root). The specific constant $\alpha$ comes from the coefficient of $q^{9}$ in $Q_5(q)^\delta$. For $\delta =\alpha$, this coefficient vanishes and changes its sign locally as $\delta$ traverses that point.

### A.3 Base ${\varvec{q}}^{\varvec{6}}$

Here we consider the infinite product

$$\begin{aligned} Q_6(q)=(q,q^5;q^6)_\infty . \end{aligned}$$

#### Conjecture 19

The *q*-series coefficients of $Q_6(q)^\delta$ exhibit the alternating sign pattern $(+-)^3$ for all $\delta \ge 3$.

Since $Q_6(-q)=(-q,-q^5;q^6)_\infty$, it is clear that Conjecture 19 is true for all positive integers $\delta$.

### A.4 Base ${\varvec{q}}^{\varvec{7}}$

Here we consider the infinite Borwein product

$$\begin{aligned} G_7(q)=\frac{(q;q)_\infty }{(q^7;q^7)_\infty }. \end{aligned}$$

#### Conjecture 20

The *q*-series coefficients of $G_7(q)^\delta$ exhibit the sign pattern $+--\,0\,0+0$ for $\delta =1$. (The zeroes indicate vanishing.) For $2\le \delta < 3$ they exhibit the sign pattern $+--+++-$, for $\delta =3$ the sign pattern $+-0+0\,0\,-$, and for $3<\delta \le 5$ the sign pattern $+-++---$.

We would like to emphasize that the periodicity of the signs of the Borwein coefficients (for any prime *p*) was already proved by Andrews [1], corresponding to the case $\delta =1$ in the conjecture. We further notice that we already proved the vanishing of the respective coefficients in the case $\delta =3$ in Corollary 13.

### A.5 Base ${\varvec{q}}^{\varvec{8}}$

The infinite product

$$\begin{aligned} Q_8(q)=\frac{(q,q^7;q^8)_\infty }{(q^3,q^5;q^8)_\infty } \end{aligned}$$

is the well-known product for the Göllnitz–Gordon continued fraction. It was shown by Hirschhorn [12] that the coefficients of $Q_8(q)$ exhibit the sign pattern $+-0+-+0-$, and that the coefficients of $Q_8(q)^{-1}$ exhibit the sign pattern $+++0---0$.

We conjecture that even more is true:

#### Conjecture 21

The *q*-series coefficients of $Q_8(q)^\delta$ exhibit for $\delta =2$ the length 16 sign pattern $+-++-+--+--+-++-$. For

$$\begin{aligned} 2.664479110226972\ldots \approx \beta \le \delta \le 4 \end{aligned}$$

they exhibit the sign pattern $+-++-+--$. (Here $\beta$ is the unique real root of the polynomial

$$\begin{aligned}&x^{12}-90x^{11}+ 1457 x^{10} + 30486 x^9 - 537081 x^8\\*&+ 1892346 x^7-{} 3683653 x^6 - 837509646 x^5 + 774767020 x^4\\*&+{} 3333687384 x^3- 40887173664 x^2 + 94379731200 x+49816166400 \end{aligned}$$

that satisfies $2<\beta <3$.) For

$$\begin{aligned} -1< \delta \le \frac{7-\sqrt{73}}{2}\approx -0.77200187265877\ldots \end{aligned}$$

they exhibit the sign pattern $+++----+$. For $\delta =-2$ they exhibit the length 16 sign pattern $+++++----+++----$.

The constant $\frac{7-\sqrt{73}}{2}$ comes from the coefficient of $q^{4}$ in $Q_8(q)^\delta$, which contains $\delta ^2-7\delta -6$ as a factor (of which $\frac{7-\sqrt{73}}{2}$ is a root). The specific constant $\beta$ comes from the coefficient of $q^{14}$ in $Q_8(q)^\delta$. For $\delta =\beta$, this coefficient vanishes and changes its sign locally as $\delta$ traverses that point.

### A.6 Base $\varvec{q}^{\varvec{1}\varvec{0}}$

Here we consider the infinite product

$$\begin{aligned} Q_{10}(q)=\frac{(q,q^9;q^{10})_\infty }{(q^3,q^7;q^{10})_\infty }. \end{aligned}$$

#### Conjecture 22

The *q*-series coefficients of $Q_{10}(q)^\delta$ exhibit for $\delta =1$ the sign pattern $+-++--+--+$, and for $\delta =-1$ the sign pattern $++++-----+$.

### A.7 Base $\varvec{q}^{\varvec{1}\varvec{1}}$

Here we consider the infinite Borwein product

$$\begin{aligned} G_{11}(q)=\frac{(q;q)_\infty }{(q^{11};q^{11})_\infty }. \end{aligned}$$

#### Conjecture 23

The *q*-series coefficients of $G_{11}(q)^\delta$ exhibit for $\delta =1$ the sign pattern $+--\,0-+\,0+0\,0\,0$, for

$$\begin{aligned} 1.7584535519419\ldots \approx \gamma \le \delta \le 2 \end{aligned}$$

they exhibit the sign pattern $+--+++-+--+$. (Here $\gamma$ is the unique real root of the polynomial

$$\begin{aligned}&x^{18}-605 x^{17}+ 157086 x^{16} - 23170380 x^{15} + 2166947862 x^{14}\\*&- 135855285510 x^{13} + 5889093658432 x^{12} - 179555226371060 x^{11}\\*&+ 3882606726301473 x^{10} - 59646447279831765 x^9 + 648313198119620778 x^8\\*&- 4932359196939174840 x^7 + 25753067609579704864 x^6\\*&- 89277087875773607120 x^5 + 194830259522753020704 x^4\\*&- 246159139789631646720 x^3 + 159155369289255052800 x^2\\*&- 42300952112982528000 x +3243869344235520000 \end{aligned}$$

that satisfies $1.5<\gamma <2$.) For $\delta =3$ the coefficients exhibit the sign pattern $+-0+-\,0-0\,0\,0\,+$. (Again, zeroes indicate that the respective coefficients vanish.)

Notice that we already proved the vanishing of the respective coefficients in the case $\delta =3$ in Corollary 13.

The specific constant $\gamma$ comes from the coefficient of $q^{21}$ in $G_{11}(q)^\delta$. For $\delta =\gamma$, this coefficient vanishes and changes its sign locally as $\delta$ traverses that point.

### A.8 Base ${\varvec{q}}^{{\varvec{1}}{\varvec{2}}}$

Here we consider the infinite product

$$\begin{aligned} Q_{12}(q)=\frac{(q,q^{11};q^{12})_\infty }{(q^5,q^7;q^{12})_\infty }. \end{aligned}$$

#### Conjecture 24

The *q*-series coefficients of $Q_{12}(q)^\delta$ exhibit for $\delta =1$ the sign pattern $+-+\,0-+-+-0+-$, for $2\le \delta \le 3$ they exhibit the sign pattern $+-+--+-+-++-$. For $\delta =-1$ they exhibit the sign pattern $+++++\,0-----0$, and for $-1<\delta <0$ the sign pattern $+++++------\,+$.
